# The subventricular zone structure, function and implications for neurological disease

**DOI:** 10.1016/j.gendis.2024.101398

**Published:** 2024-08-26

**Authors:** Kaishu Li, Yin Zheng, Shubing Cai, Zhiming Fan, Junyi Yang, Yuanrun Liu, Shengqi Liang, Meihui Song, Siyuan Du, Ling Qi

**Affiliations:** a Department of Neurosurgery, The Affiliated Qingyuan Hospital (Qingyuan People's Hospital), Guangzhou Medical University, Qingyuan, Guangdong 511518, China; b Institute of Digestive Diseases, The Affiliated Qingyuan Hospital (Qingyuan People's Hospital), Guangzhou Medical University, Qingyuan, Guangdong 511518, China

**Keywords:** Glioblastoma multiforme, Neural injury repair, Neural stem cells, Neurodegenerative, Subventricular zone

## Abstract

The subventricular zone (SVZ) is a region surrounding the lateral ventricles that contains neural stem cells and neural progenitor cells, which can proliferate and differentiate into various neural and glial cells. SVZ cells play important roles in neurological diseases like neurodegeneration, neural injury, and glioblastoma multiforme. Investigating the anatomy, structure, composition, physiology, disease associations, and related mechanisms of SVZ is significant for neural stem cell therapy and treatment/prevention of neurological disorders. However, challenges remain regarding the mechanisms regulating SVZ cell proliferation, differentiation, and migration, delivering cells to damaged areas, and immune responses. In-depth studies of SVZ functions and related therapeutic developments may provide new insights and approaches for treating brain injuries and degenerative diseases, as well as a scientific basis for neural stem cell therapy. This review summarizes research findings on SVZ and neurological diseases to provide references for relevant therapies.

## Introduction

The mammalian brain, a complex organ of remarkable plasticity, harbors regions of ongoing neurogenesis throughout life. One such region, the subventricular zone (SVZ), is a thin layer of tissue (approximately 0.1–3 mm thickness), located around the lateral ventricles.^1^ As one of the most active neurogenic zones in the adult mammalian brain, the SVZ is a reservoir of neural stem cells (NSCs) and neural progenitor cells (NPCs) that continuously proliferate and differentiate into various types of neurons and glial cells.^2^

The cellular composition and distribution within the SVZ are characterized by complexity and heterogeneity, encompassing diverse types of NSCs and NPCs.^3^ These cells play pivotal roles in the onset and progression of neurological disorders. Emerging evidence suggest that functional abnormalities of SVZ cells are associated with a variety of neurological diseases, including brain tumors, Parkinson's disease (PD), Alzheimer's disease (AD), and Huntington's disease (HD).

Understanding the anatomical location, structure, cellular composition, physiological functions of SVZ cells, and their relationship with diseases is of paramount importance for elucidating the mechanisms of neural development and diseases.^4^ Moreover, it holds significant implications for the development of stem cell therapy.^5^ A deeper exploration and understanding of the complex biological functions of SVZ could provide a foundation and reference for neural stem cell therapy, offering new insights and approaches for the treatment and prevention of neurological diseases.^6^

In light of recent reports and advancements in the field, this study aims to further investigate the role of SVZ cells in neurological disorders, with the ultimate goal of contributing to the development of effective therapeutic strategies.

### Structure and cellular composition of the SVZ

#### Healthy human SVZ

**Figure 1:**
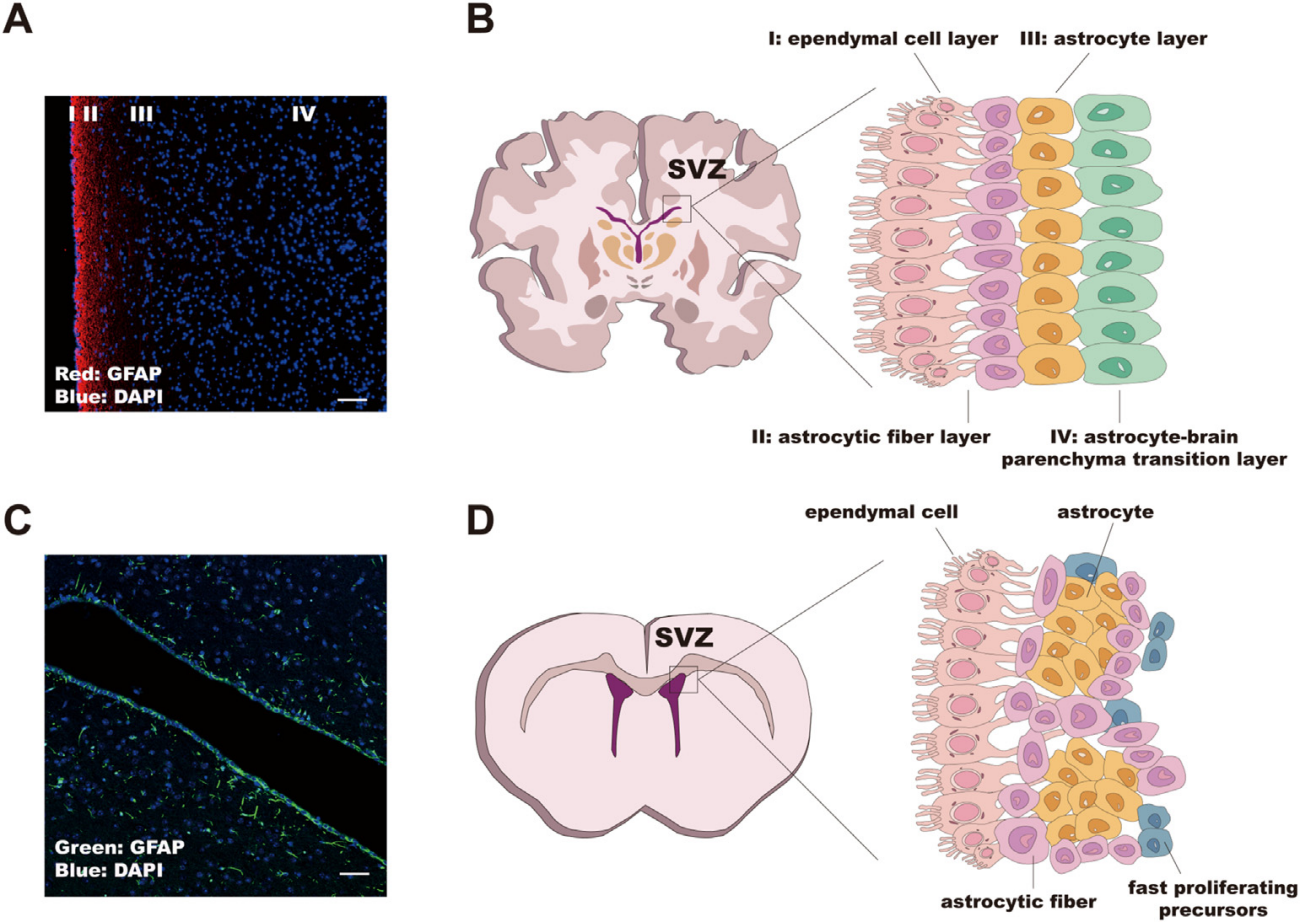
Comparison of subventricular zone (SVZ) cellular composition between human glioblastoma multiforme (GBM) patients and rodent models. **(A)** Regions of the SVZ partitioned based on surgical samples from GBM patients. **(B)** Schematic diagram of the different cellular compositions of human SVZ. **(C)** Cellular dynamics in the SVZ of rodent models revealed by immunofluorescence staining. **(D)** Schematic diagram of the different cell composition of the SVZ of rodent models.

The formation of the SVZ commences during the early developmental stages of the embryonic neural tube, where NSCs originate from the walls of the neural tube and begin to migrate toward the neural tube cavity. As embryonic development progresses, the ependymal cell layer gradually forms, eventually giving rise to the ventricular system. The SVZ is a thin, band-like structure located beneath the corpus callosum, encircling the lateral wall of the lateral ventricle, primarily composed of four characteristic cell layers. From the inside out, these are the ependymal cell layer (Layer I), the astrocytic fiber layer (Layer II), the astrocyte layer (Layer III), and the astrocyte-brain parenchyma transition layer (Layer IV).^7^ The cellular composition of the human SVZ is shown in Figure 1A and B.

Ependymal cells originate from radial glial cells, undergoing multiple cell differentiations to eventually form a single layer of columnar epithelial cells covering the lateral wall of the lateral ventricle with cilia, separating the brain parenchyma from the lateral ventricle.^8^, ^9^, ^10^ The cilia on the surface of the ependymal cells exhibit distinct planar polarity, with their bases oriented towards the direction of cerebrospinal fluid flow, thus aiding in the circulation of cerebrospinal fluid. Based on the number of cilia, ependymal cells can be further subdivided into two types, E1 and E2.^11^ E1 cells possess multiple long cilia and several small basal bodies, while E2 cells exhibit a biflagellate appearance with two large and complex basal bodies. Previous views held that ependymal cells lacked the potential of NSCs, but recent research suggests that they may be dormant NSCs, as they can regain NSC functionality under specific stimuli, such as striatal injury, basic fibroblast growth factor (bFGF), or vascular endothelial growth factor (VEGF).^12^, ^13^, ^14^ Overall, ependymal cells play a crucial role in cerebrospinal fluid circulation and NSC regeneration.^15^

Layer II of the SVZ, adjacent to Layer I, lacks cellular components and is primarily composed of dense astrocytic synapses, with a few neuronal bodies and astrocytes.^16^ The biological function of Layer II in humans remains unclear, but it may regulate neuronal function and control the proliferation and differentiation of NSCs during development through the interconnections between the dominant astrocytes and ependymal cells in this layer.^17^^,^^18^

Layer III, first reported by Sanai et al in 2004, is the layer with the highest cell density and is primarily composed of closely connected astrocytes.^19^ It is characterized by the expression of glial fibrillary acidic protein (GFAP) and SRY-box 2 (Sox 2). Furthermore, electron microscopy analysis reveals the presence of a few oligodendrocytes and displaced ependymal cells in this layer, where the cilia of the ependymal cells do not face the ventricle but form cell clusters similar to the ependymal layer with surrounding cells.^20^ Astrocytes, the most common type of glial cells in the central nervous system (CNS), participate in the regulation of neuronal function by releasing various signaling molecules and cytokines, including glutamate, γ-aminobutyric acid (GABA), interleukin-1β (IL-1β**)**, interleukin-6 (IL-6), and by engulfing neuronal synaptic vesicles and regulating neuronal background discharge.^21^^,^^22^ Research has shown that Layer III contains the majority of NSCs and NPCs within the SVZ, emphasizing their crucial role in neurogenesis.^23^ This finding highlights the importance of these cells in Layer III for the continuous processes of brain development and plasticity.^24^^,^^25^ NSCs/NPCs are vital for brain repair and regeneration. After injury or disease, these cells can migrate from the SVZ to the damaged site, where they can proliferate and differentiate into specific neural cell types, thereby replacing damaged neurons or contributing to tissue repair mechanisms.^26^^,^^27^

Layer IV, characterized by the presence of myelinated cells, serves as a transition zone between astrocytes and brain parenchyma.^28^^,^^29^ The biological function of this layer remains largely undefined.^30^

### Differences between human SVZ and rodent SVZ

In the realm of SVZ research, it is important to note the differences in structure and function between the human SVZ and that of commonly used animal models, such as rodent models. Therefore, it is necessary to describe the SVZ of rodents as well. To date, several key differences have been noted in comparing the structure and cellular composition of the SVZ between human and rodent models. In humans, the SVZ is larger and more dispersed compared with rodents, where it is more extensive and prominent.^31^ The organization of the SVZ in humans allows for a larger pool of neural stem/progenitor cells.^32^ Additionally, the cellular composition varies, with differences in the abundance and distribution of NSCs, progenitor cells, and other cell types such as ependymal cells and astrocytes.^16^^,^^32^ The cellular composition of the rodent models is shown in Figure 1C and D. While both human and rodent SVZ contain NSCs, transit-amplifying progenitor cells, and migrating neuroblasts, the proportion and distribution of these cell types differ between species.^16^^,^^33^ Human SVZ exhibits a greater diversity of cell types and subtypes, reflecting the complexity of human brain development and function.^19^ The structural and cellular characteristics of the SVZ undergo age-related changes in both humans and rodents, but the extent and consequences of these changes differ between species. Human SVZ aging is associated with alterations in stem cell dynamics, neurogenic capacity, and niche architecture, which may contribute to age-related cognitive decline and neurodegenerative diseases such as AD.^16^ This difference in neurogenic capacity may be related to the larger size and complexity of the human brain. The microenvironment or niche surrounding the SVZ cells also differs between humans and rodents.^34^ Human SVZ niches exhibit unique signaling molecules and extracellular matrix (ECM) components that regulate stem cell behavior and neurogenesis, influencing cell fate determination and migration patterns.^35^ Studies have revealed differences in gene expression profiles between human and rodent SVZ cells, suggesting species-specific regulatory mechanisms underlying neurogenesis and gliogenesis.^36^ These differences may contribute to variations in cell fate determination, proliferation, and differentiation processes.^37^

Recent discoveries on the structural organization of dura mater highlight the critical roles of membranous structures in the CNS.^38^ The SVZ, as an important membranous structure in the CNS, contains much more diverse cell populations than dura mater, yet its precise functions remain poorly defined with many controversies. For instance, Li et al showed that glioblastoma multiforme (GBM) rarely disrupts the SVZ and disseminates into the cerebrospinal fluid clinically, proposing that the Layer I of SVZ (the ependymal layer) may block GBM invasion into the lateral ventricles.^39^ However, Norton argued that GBM is capable of physically disrupting the ependymal barrier in animal models, leading to compromised ependymal integrity and increased interactions between cerebrospinal fluid and tumor volumes.^40^ We reason that structural differences likely exist between human and rodent SVZ,^41^ which may not fully recapitulate the uniqueness of the human SVZ. Collectively, these findings suggest that further investigations are required to better delineate the structural organization and functional properties of the human SVZ.

### The role of SVZ in neurodegenerative diseases

#### The significance of SVZ in neural injury repair

CNS regeneration is critical for maintaining brain homeostasis. For long, the scientific community believed the adult mammalian CNS (particularly the brain and spinal cord) lacks regenerative capacity, as regeneration was rarely observed following injury, and methods to effectively promote regeneration were lacking.^42^ However, evidence now shows the CNS does have some capacity for self-repair and regeneration under certain conditions, as revealed by precise imaging techniques, the discovery of endogenous NSCs, growth factors promoting repair, and other advances.^43^ Nonetheless, current research strategies cannot yet achieve full or near-normal restoration of neural circuits.^44^ Exploring feasible strategies to promote repair after neural injury thus remains a focus in brain research.

Neural injury repair involves multiple mechanisms such as stem cell differentiation, neuronal regeneration, and immune response, which interact to restore neural network function. Among them, neuronal regeneration and stem cell differentiation are the most important mechanisms, which can replace damaged neurons and glial cells and promote the reconstruction and recovery of neural networks.^45^

Current research indicates that NSCs, NPCs, and ependymal cells in the SVZ all possess the potential for neural repair. They participate in the process of neural injury repair through various mechanisms such as differentiation into neurons and glial cells, secretion of various neurotrophic factors and growth factors, and regulation of peripheral inflammation.^45^^,^^46^ Meanwhile, factors such as blood vessels, ECM, and transcription factors also play an important regulatory role in neural repair in the SVZ. Under the stimulation of injury factors, these cells can differentiate into neurons and glial cells, replacing damaged neurons and glial cells and promoting the reconstruction and recovery of neural networks. They can also secrete various neurotrophic factors and growth factors, such as nerve growth factor (NGF),^47^ brain-derived neurotrophic factor (BDNF), and fibroblast growth factor (FGF), and activate multiple signaling pathways such as PI3K (phosphoinositide 3-kinase)/Akt (protein kinase B), ERK (extracellular signal-regulated kinase)/MAPK (mitogen-activated protein kinase), Wnt/β-catenin, Notch, FGF2, to promote the regeneration of injured neurons and glial cells, inhibit inflammation and apoptosis, and promote neuronal growth.^45^^,^^48^, ^49^, ^50^

Previously, it was believed that ependymal cells did not possess stem cell functions, but current research suggests that under special circumstances, ependymal cells can be activated by injury factors and participate in the neural repair process by secreting various cell factors and signaling molecules.^51^ For example, cell factors such as bone morphogenetic protein signaling regulators, pigment epithelium-derived growth factors, and matrix cell-derived factor 1 can regulate adult neurogenesis and determine the size of the NSCs pool. In addition, ependymal cells can respond to the necrosis of ependymal cells by replacing damaged cells. Recent studies have shown that ependymal cells can also regulate the growth and differentiation of neurons by releasing extracellular vesicles carrying microRNAs.

The ECM and blood vessels in the SVZ also play an irreplaceable role in the process of neural injury repair. The stem cell niche in the SVZ is located in an ECM environment composed of glycoprotein tenascin-C (TNC) and laminin (LN).^52^ In the SVZ, this special ECM environment can regulate adult neurogenesis through direct contact with NSCs. The ECM regulates the differentiation, proliferation, and migration of neurons and glial cells through interaction with NSCs and NPCs, participating in neural injury repair.^53^ For example, ECM-related heparan sulfate proteoglycan binds to growth factors to regulate the proliferation of NSCs in the SVZ. In addition, studies have shown that blocking the expression or function of LN in NSCs can inhibit the migration and differentiation of NSCs, thereby affecting the process of neural injury repair.

Furthermore, Gli family zinc finger 1 (Gli 1) and Gli family zinc finger 2 (Gli 2) are key transcription factors in the differentiation process of NSCs. They play important roles in regulating cell proliferation, differentiation, and stem cell self-renewal, and can drive NSCs to differentiate into different types of neurons at different locations. In driving the transcription process of NSCs, Gli1 and Gli2 mainly function by regulating the activity of the Wnt and Sonic hedgehog (Shh) signaling pathways. On the one hand, Gli1 and Gli2 can promote the activation of the Shh signaling pathway, thereby stimulating the proliferation and self-renewal of NSCs. On the other hand, they can regulate the activity of the Wnt signaling pathway, promoting the differentiation of NSCs into neurons or neuroglial cells. The functions of Gli1 and Gli2 interact with other transcription factors and signaling pathways, forming a complex regulatory network, thereby affecting the differentiation outcome of NSCs.^54^

In conclusion, NSCs, NPCs, and ependymal cells in the SVZ all have the potential for neural repair. They participate in repair after neural injury by differentiating into neurons and glia, secreting various neurotrophic factors and growth factors, and regulating peripheral inflammation.^55^ However, NSC research is still an emerging field with many issues to be addressed. How to stably and abundantly induce the proliferation of NSCs in the SVZ and direct their migration to injured sites for integration with the damaged neural system remains an important future research direction for SVZ-based therapy of neural system injuries.

### The correlation between SVZ and neurodegenerative diseases

Neurodegenerative diseases represent a class of chronic progressive neurological disorders characterized by neuronal degeneration and death. Although different neurodegenerative diseases have distinct pathogenic mechanisms, recent studies suggest that impaired adult neurogenesis is a common feature in the early stages of neurodegenerative diseases such as AD and PD. In these conditions, the quantity and function of NSCs in the SVZ may be affected, leading to reduced or interrupted neurogenesis.^56^, ^57^, ^58^

### The role and therapeutic potential of SVZ in AD

In AD, the deposition of β-amyloid (Aβ) oligomers induces a reduction in the proliferation of NSCs in the SVZ, thereby leading to decreased neurogenesis.^59^ Some studies suggest that Aβ inhibits neurogenesis in the SVZ by regulating the cell cycle, including up-regulating the expression of CDC20 homologue 1 (Cdh1) and down-regulating the expression of cyclin-dependent kinase 5 (CDK5)/p35 and cyclin B1 (CCNB1).^60^^,^^61^ Additionally, the abnormal aggregation of tau protein is associated with the onset of neurodegenerative diseases. Tau protein, involved in many cellular functions, regulates the stability of the cytoskeleton in neurogenesis. In AD, abnormally phosphorylated tau protein reduces microtubule binding capacity, leading to microtubule instability and reduced cell proliferation in the SVZ.^62^^,^^63^ On the other hand, NSCs within the SVZ may serve as potential therapeutic targets for neurodegenerative diseases. Research on related therapies has primarily focused on the inhibition of NSCs within the brain, with direct targeting of the SVZ region to promote NSC generation being relatively rare. Several animal studies involving the transplantation of human or murine-derived NSCs/NPCs into AD mouse models have demonstrated that stem cell transplantation can improve cognitive and memory functions in mice. It is noteworthy that due to the relatively limited sources of NSCs, some researchers are exploring cell transplantation using dopaminergic neurons derived from pluripotent stem cells to replace diseased neurons, with promising results shown in animal models. Additionally, the transplantation of mesenchymal stem cells derived from umbilical cord blood appears to aid in improving spatial learning abilities in AD mouse models. At present, although the aforementioned stem cell therapies have shown exciting prospects in animal models, their effectiveness in human AD patients remains limited. Further exploration through additional animal experiments and clinical trials is necessary.^64^, ^65^, ^66^

### The role and therapeutic potential of SVZ in PD

PD is a movement disorder characterized by bradykinesia, rigidity, and resting tremors. It is the second most common neurodegenerative disease, following AD.^67^ The typical neuropathological features of PD include the gradual loss of dopaminergic neurons and the dysregulation of astrocyte and microglial activation.^68^ Current research suggests that chronic neuronal deterioration in the PD brain may be associated with aging, increased neuroinflammation and oxidative stress, and dysfunction of organelles such as mitochondria.^69^, ^70^, ^71^ In PD, due to dopamine depletion, neurogenesis in the SVZ is reduced. In the CNS, the Wnt signaling pathway is responsible for regulating and coordinating various aspects of neuronal function, with the Wnt/β-catenin pathway being extensively studied.^72^ Research indicates that dysfunctional Wnt/β-catenin signaling is a key factor in the decline of neurogenesis in the SVZ in PD. During the progression of PD, age-dependent oxidative stress and inflammatory pathways are associated with down-regulation of Wnt/β-catenin signaling in NSC niches and significant up-regulation of endogenous Wnt antagonists, thereby affecting neurogenesis in the SVZ.^73^ Astrocytes and microglia are important sources of endogenous Wnt ligands, and dysregulated activation of astrocytes and microglia in PD leads to a decrease in Wnt ligands, thereby affecting neurogenesis.^74^ The specific mechanism involves the gradual down-regulation of Wnt signaling during aging, the up-regulation of endogenous secreted Fzd-related protein (sFRP) and Dkk family Wnt antagonists, and the overexpression of the key β-catenin destruction complex protein glycogen synthase kinase 3β (GSK-3β). The combination of key risk factors, especially inflammation and 1-methyl-4-phenyl-1,2,3,6-tetrahydropyridine (MPTP) exposure, collectively impair Wnt signaling both inside and outside the neurogenic niche, adversely affecting adult neurogenesis in the SVZ and nigrostriatal neuronal repair.^75^ Furthermore, research suggests that dopamine stimulates the release of epidermal growth factor (EGF) through the PKC (protein kinase C) pathway, thereby initiating various intracellular responses and ultimately promoting the selective expansion of stem/progenitor cells.^76^ This finding suggests that by inducing the proliferation and differentiation of NSCs in the SVZ, it may be possible to help repair damaged dopaminergic neurons and thus alleviate the symptoms of PD.

### The role and therapeutic potential of SVZ in HD

HD is an autosomal dominant hereditary neurodegenerative disease caused by a substantial expansion of CAG repeat sequences in the Huntington protein (HTT) gene. Its prominent feature is the gradual loss of spiny neurons in the striatum, with other regions potentially affected in late-stage patients.^77^^,^^78^ Clinically, it manifests as chorea, psychiatric disorders, and progressive dementia, known as the triad. In HD, an increased thickness of the niche and enhanced proliferation of resident cells in the SVZ are observed, with widespread destruction of lipid structures, particularly in the myelin phospholipid layer. Studies have reported that the degradation of the myelin phospholipid layer may lead to a shift in myelin metabolism, thereby promoting neurogenesis.^79^ Like most neurodegenerative diseases, HD lacks specific treatment methods. Current treatments are mainly symptomatic, including antagonistic dopaminergic drugs and dopamine receptor inhibitors, increasing the content of acetylcholine, increasing the content of γ-aminobutyric acid in the CNS, treatment of psychiatric disorders, as well as cell transplantation.

### Application of SVZ cells in the treatment of neurodegenerative diseases

Neurodegenerative diseases include AD, PD, and HD, all of which are caused by neuronal death and synaptic loss. SVZ cells can play a role in several ways, providing an important cell source and treatment strategy for the treatment of neurodegenerative diseases. However, these treatment plans are still in the laboratory and clinical trial stages and require further research and verification. The application of SVZ cells in the treatment of neurodegenerative diseases mainly includes the following aspects.

### NSC transplantation

NSCs in the SVZ can be used for the treatment of neurodegenerative diseases through transplantation. These cells can differentiate into various cell lineages, including neurons, astrocytes, and oligodendrocytes.^80^ They have strong self-renewal capabilities and can produce a large number of progeny cells. Using them as cell replacement therapy can help compensate for the loss of neurons in the brains of patients with neurodegenerative diseases, making them ideal cell donors for the treatment of these diseases, thereby promoting the repair and regeneration of neural tissue.^81^ At present, research on NSC transplantation as a therapeutic intervention remains predominantly in the preclinical phase. In the context of PD, investigations have revealed that NSC transplantation, when combined with ethyl stearate, can up-regulate the expression of C–C motif chemokine ligand 5 (CCL5) and C–C motif chemokine receptor 5 (CCR5) within the brain. This combination therapy facilitates the migration of NSCs from the striatum to the substantia nigra, their differentiation into dopaminergic neurons, and the enhancement of motor behavior in PD rat models.^82^ Yuan et al discovered that overexpression of nuclear receptor-related factor 1 (Nurr1) in both NSCs and microglia, followed by their co-transplantation, could ameliorate the cerebral milieu in rats by attenuating the release of pro-inflammatory cytokines, offering significant therapeutic promise for PD.^83^ In AD, NSC transplantation has been shown to safeguard basal forebrain cholinergic neurons, repair synaptic deficits, and augment the expression of cognitive-related proteins within the hippocampus, culminating in improved learning and memory functions in AD mouse models.^84^ McGinley et al have validated the targeting of the corpus callosum with a genetically engineered human NSC line in an AD mouse model, ascertaining that a subcutaneous dose of 3 mg/kg tacrolimus is the minimal immunosuppressive dosage necessary for the survival of these modified NSCs.^85^ The introduction of human NSCs into the striatum of HD zQ175 mice has been demonstrated to elevate BDNF levels and mitigate the accumulation of mutant Huntington protein (mHTT), leading to ameliorated behavioral impairments.^86^ Despite the considerable potential of NSC transplantation in treating neurodegenerative disorders, its clinical application is hindered by the propensity of the transplanted NSCs to differentiate predominantly into glial cells, coupled with their limited migratory capacity, suboptimal viability, and susceptibility to immune rejection.^87^ Furthermore, the disparities in brain structure and function between the aforementioned animal models and humans, as well as the structural and functional variations of the SVZ, may also contribute to the challenges encountered in current research, given the greater complexity of the human brain's architecture and its functionalities. In addition, the large number of cells required for NSC transplantation makes cell sourcing a primary issue that must be resolved for the clinical application and scientific research of NSC transplantation.

### Cytokine therapy

Cells within the SVZ secrete an array of cytokines and trophic factors, such as stromal-derived factor 1 (SDF-1), ciliary neurotrophic factor (CNTF), leukemia inhibitory factor (LIF), and IL-6. These substances are influential in modulating neuronal growth, differentiation, and migration. Notably, SDF-1 expression within the SVZ is markedly variable, with elevated levels in the ependymal layer potentially inducing neurogenic quiescence, whereas reduced levels may enhance NSC proliferation and differentiation.^88^ Additionally, the targeted intracerebral delivery of matrix-derived factor SDF-1α, which adjusts its brain concentration, has been shown to facilitate the migration of intravenously administered NSCs to the CNS.^89^ CNTF, produced by SVZ astrocytes, triggers intrinsic neuroprotective responses *in vivo*, offering protection against the progressive loss of dopaminergic neurons and ameliorating motor deficits in PD models such as the MPTP and α-synucleinopathies.^90^ Reactive microgliosis within the SVZ, in response to CNS injury, has been implicated in the release of pro-inflammatory cytokines (IL-6, IL-1β, tumor necrosis factor alpha (TNF-α)), which contribute to neuronal dysfunction and degeneration. These inflammatory mediators may adversely impact the NSC niche, diminishing neuronal proliferation and differentiation, and consequently, neurogenesis.^91^ Upon activation of endogenous neural repair mechanisms, neurogenic cytokines such as LIF, CNTF, and IL-6 are released, albeit their role in neural repair remains constrained.^92^ Overall, future cytokine therapy for neurodegenerative diseases should aim to harness the beneficial effects of cytokines while neutralizing the detrimental ones, thereby promoting axonal regeneration, neural tissue remodeling, and functional restoration, ultimately contributing positively to the repair of neural tissue in neurodegenerative conditions.

### Directed migration and survival of neurons

Newly born neurons in the SVZ can replace lost neurons in damaged neural tissue areas through a series of directed migration and differentiation and survive. For newly born neurons in the SVZ, they first need to differentiate into the appropriate neuronal subtype with high fidelity. The most challenging part is that after transplantation, they need to project to the appropriate target neurons and form appropriate synaptic connections, participating in the formation and repair of neural networks.^93^ Currently, for PD, we need to transplant NSCs into the striatum, while other neurodegenerative diseases show the loss of different neuronal subtypes in multiple brain regions, making the directed migration and survival of SVZ neurons more challenging.^57^^,^^94^

### The relationship and therapeutic potential between SVZ and gliomas/GBM

#### SVZ is closely related to the occurrence and development of GBM

**Figure 2:**
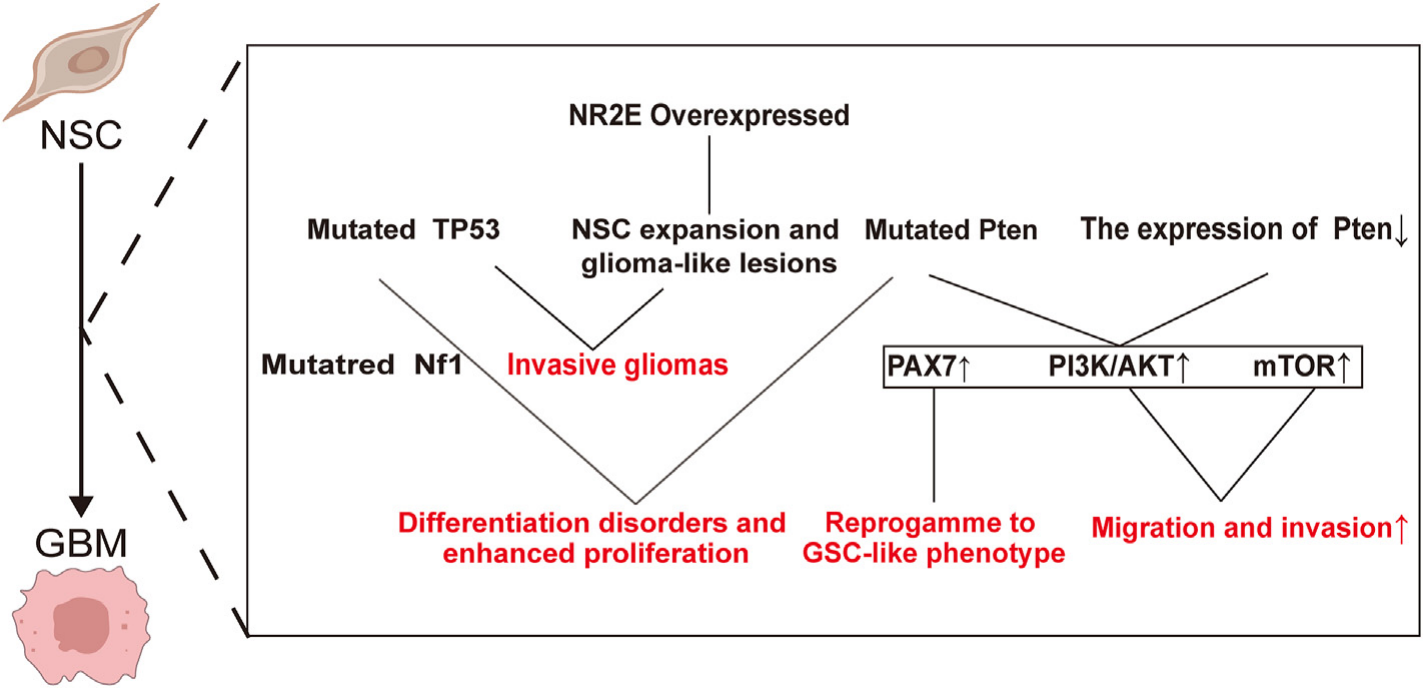
The relationship between subventricular zone (SVZ) cells and glioblastoma multiforme (GBM) development.

**Figure 3:**
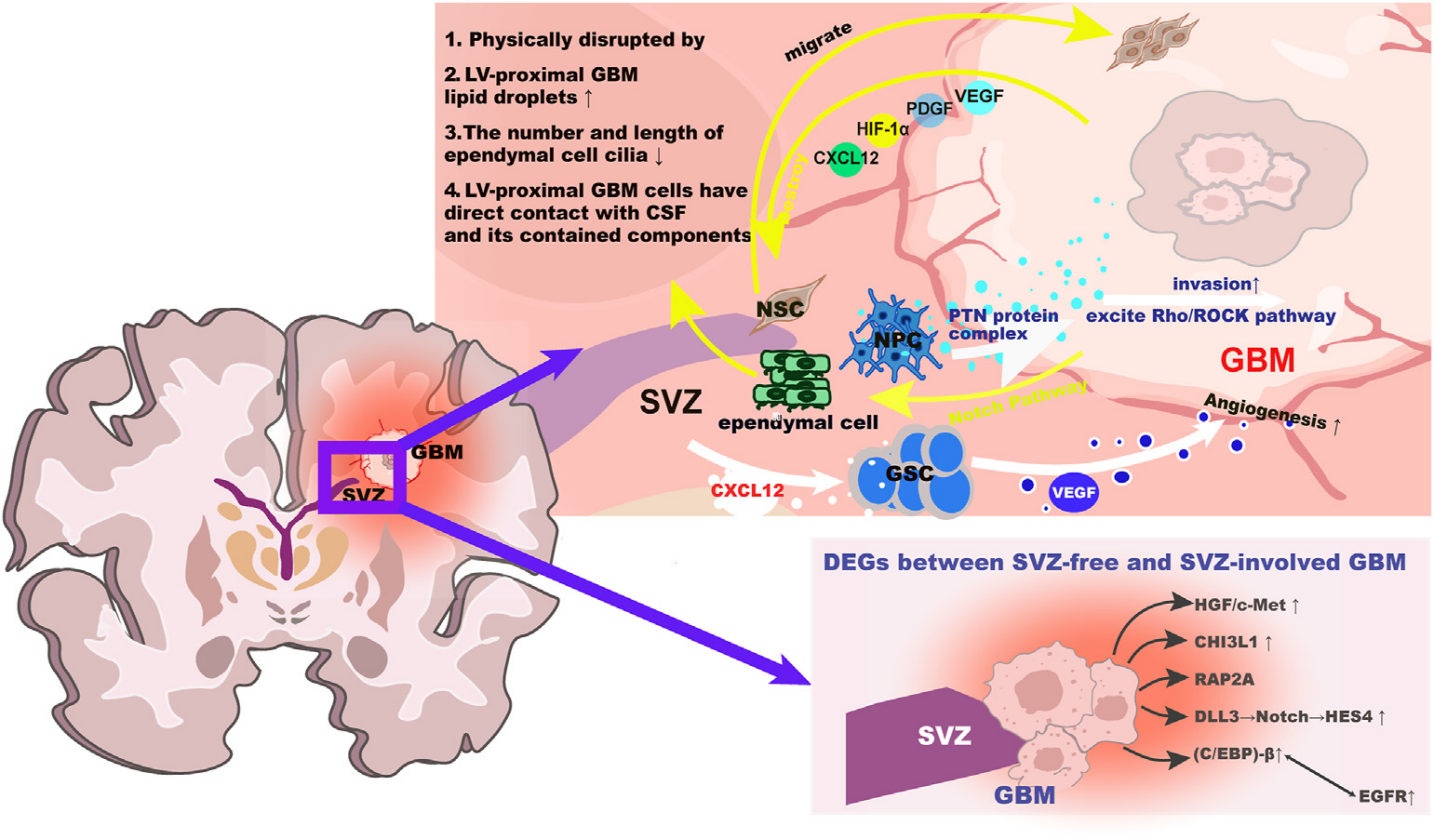
The relationship between subventricular zone (SVZ) cells and glioblastoma multiforme (GBM) progression. SVZ affects GBM through secretions and signaling pathways (yellow scissors) and promotes its invasion and growth by secreting factors (white scissors).

Gliomas are the most common primary tumors of the nervous system. According to the latest definition by the WHO, gliomas are divided into grades 1 to 4 from low to high based on pathological morphological features and molecular expression characteristics.^95^ Among them, grade 4 gliomas with wild-type isocitrate dehydrogenase (IDH1) are defined as GBM, which are the most common and malignant gliomas. GBM has a poor prognosis and is prone to early recurrence.^96^ The most in-depth research is on the correlation between SVZ and GBM, and it has been confirmed that SVZ has an extremely complex relationship with the occurrence, development, recurrence, and treatment resistance of gliomas, mainly GBM.^97^ The occurrence and development processes of SVZ's participation in GBM are shown in Figure 2, Figure 3.

### The relationship between human SVZ cells and GBM occurrence and development

The proliferation, migration, and differentiation of SVZ NSCs into NPCs, culminating in the maturation into neurons, play a crucial role in neurogenesis.^98^ NSCs, due to their role in neurogenesis, are hypothesized to be the cells of origin for GBM.^19^ Additionally, primary GBM-associated epidermal growth factor receptor (EGFR) amplification and phosphatase and tensin homolog (PTEN) loss deleted on chromosome ten, secondary GBM-associated tumor protein p53 (TP53) inactivation, PTEN loss, and telomerase reverse transcriptase (TERT) mutations, are all involved in the control of NSCs.^99^, ^100^, ^101^, ^102^, ^103^ Lee et al found that astrocyte-like NSCs with driver mutations spread from the SVZ, leading to GBM development in distal brain regions, providing direct genetic evidence for the theory that NSCs are the origin cells for GBM.^104^ In the following text, we will discuss some genes, factors, and pathways related to GBM origin in SVZ NSCs. The tumor suppressor gene TP53 in the SVZ integrates stress signals from damaged cells, promoting cell cycle arrest and apoptosis, thereby blocking the transmission of DNA mutations.^105^ When astrocyte-like NSCs undergo TP53 mutation, the proliferation and chemotaxis of astrocyte-like B and C cells in the SVZ are accelerated, altering the niche of neurogenic cells in the SVZ. This TP53 mutation-induced focal proliferation in the SVZ, when subjected to additional stimuli, can induce GBM.^106^^,^^107^ The tumor suppressor gene PTEN, through encoding a phosphatase, regulates the migration of mouse NSCs while inhibiting their proliferation and self-renewal.^108^ Initially, PTEN mutation weakens the suppression of the PI3K/AKT and mTOR (mechanistic target of rapamycin) pathways, leading to autophagy inhibition, increased protein synthesis, and promotion of NPC migration and invasion.^109^^,^^110^ Furthermore, the simultaneous deletion of tumor suppressor genes PTEN and TP53 exacerbates differentiation disorders and proliferation in NSCs.^103^^,^^111^ PTEN loss induces up-regulation of paired box 7 (PAX7), reprogramming NSCs into a glioma stem cell phenotype.^102^^,^^112^ The oncogene rat sarcoma (Ras), via expressing activated Kirsten rat sarcoma viral oncogene (K-ras), engages pathways such as MAPK and PI3K, contributing to brain tumor cell generation, proliferation, migration, and angiogenesis.^113^, ^114^, ^115^ Moreover, K-ras can stimulate the growth of retinoblastoma gene (RB) gene-deficient astrocytes, facilitating the onset of GBM.^114^ In SVZ NSCs, promoter mutations in the TERT gene (pTERT) stabilize telomere length, fostering cell self-renewal and expansion, thereby promoting GBM formation.^112^^,^^116^

In addition, several factors and pathways are implicated in GBM onset. EGFR, predominantly expressed in type C cells in the human SVZ, regulates cell proliferation, invasion, and anti-apoptosis through pathways such as PI3K/Akt and Ras/Raf/MEK/ERK.^117^^,^^118^ Platelet-derived growth factor receptor-α (PDGFRα) modulates brain cell cycle and migration, and its abnormal activation stimulates NSC proliferation.^119^^,^^120^ The transcriptional regulator nuclear receptor subfamily 2 group E member 1 (NR2E1) binds to the promoter of the PTEN gene, inhibiting its expression and preserving NSCs' proliferative potential.^121^ Dysregulation of the Wnt pathway, involved in synaptic functions, neuronal migration, and axonal guidance, may promote the transformation of NSCs into glioma stem cells.^121^^,^^122^ Additionally, elevated expression of β-catenin and related transcription factor 4 (TCF4) in GBM tissue suggests a potential contribution of abnormal Wnt/β-catenin signaling to GBM onset.^123^

Yuan et al analyzed the differentially expressed genes between healthy SVZ and SVZ tissues invaded by GBM.^124^ Their findings revealed that the differentially expressed genes were predominantly enriched in tumor immune-related pathways, notably PI3K-Akt, NF-κB, and JAK-STAT, which regulate T cell differentiation, humoral immunity, and inflammation.^125^^,^^126^ Subsequent investigations employing mRNA array and proteomic analyses unveiled variations in the expression levels of various genes, including hepatocyte growth factor (HGF), chitinase 3 like 1 (CHI3L1), RAP2A, hairy and enhancer of split 4 (HES4), delta-like 3 (DLL3), holliday junction recognition protein (HJURP), EGFR, CCAAT enhancer binding protein β (C/EBP-β), and signal transducer and activator of transcription 3 (STAT3) between GBM tissues with and without SVZ involvement.^127^, ^128^, ^129^ Notably, the HGF/c-Met axis was found to be heightened in GBM involving the SVZ, leading to disturbances in cell cycle regulation and enhanced tumor cell proliferation.^32^^,^^127^ Additionally, the activation of mesenchymal to epithelial transition factor (MET) was observed to stimulate various pathways, including PI3K/Akt, MAPK, and STAT3, facilitating tumor cell growth and evasion from apoptosis.^126^^,^^130^^,^^131^ CHI3L1 gene expression was notably elevated in GBM involving the SVZ, correlating with disease severity, increased invasion, immune infiltration, poor prognosis, and shortened survival.^132^, ^133^, ^134^ RAP2A-dependent actin cytoskeleton remodeling was found to inhibit the invasiveness of GBM accumulating in the SVZ.^135^ Furthermore, the activation of Notch signaling in the SVZ was associated with reduced overall survival, with HES4 and DLL3 exhibiting high expression levels.^129^ Bidirectional regulation between nuclear transcription factor C/EBP-β and EGFR was observed to promote GBM proliferation and invasion in the SVZ.^136^^,^^137^ Additionally, the SVZ was found to drive GBM invasion through the secretion of various chemical factors, such as pleiotrophin (PTN) complexes and C-X-C motif chemokine 12 (CXCL12), inducing angiogenesis and providing nutrients for tumor growth.^138^^,^^139^ Moreover, glioma stem cells located in the SVZ were identified to possess specific resistance to radiation, potentially contributing to recurrence after radiotherapy.^140^ GBM secretions were also implicated in altering SVZ structure and gene expression via extracellular vesicles, as well as thickening the SVZ and increasing stem cell marker levels through Nestin and platelet-derived growth factor A (PDGF-A) expression.^141^, ^142^, ^143^

### The relationship between rodent SVZ cells and GBM occurrence and development

PTEN plays a crucial role in regulating the migration, proliferation, and self-renewal of mouse NSCs through its phosphatase activity.^108^ Mutation in PTEN initially disrupts the suppression of the PI3K/AKT and mTOR pathways, resulting in the inhibition of autophagy, increased protein synthesis, and promotion of NSC migration and invasion.^109^^,^^110^ Doetsch et al, upon injecting EGF into the lateral ventricles of mice, noted a reduction in NPC numbers, with type C cells transforming into highly invasive and proliferative neuroglial-like cells.^144^ Alcantara et al demonstrated *in vivo* mouse experiments that mutations in the tumor suppressor gene neurofibromin 1 (Nf1) lead to aberrant NSC/NPC proliferation and differentiation, laying the groundwork for GBM formation.^98^ Moreover, PDGFRα can regulate brain cell cycle and migration, and its abnormal activation stimulates NSC proliferation.^119^^,^^120^ Liu et al showed that overexpression of NR2E1 in NSCs specifically induces adult mouse NSC amplification and neuroglioma-like lesions, which, when combined with TP53 mutation, progress into invasive gliomas.^145^^,^^146^

Continuing from the previous discussion on Notch signaling's involvement in GBM pathogenesis, DLL3, a Notch receptor ligand, and HES4, a downstream target gene of Notch signaling transduction, play crucial roles. Upon activation, HES4 exerts tissue-specific transcriptional suppression, inhibiting cell differentiation and maintaining stem cell characteristics.^147^ Although the study did not delve into the potential link between HES4 and tumor cell stemness maintenance, other research suggests its involvement in controlling the proliferative properties of NSCs during retinal development.^148^ This suggests that HES4 may play a similar role in the SVZ or within tumor cells. Additionally, cerebrospinal fluid-induced serpina3 expression is associated with decreased survival expectation and enhanced GBM invasion and proliferation.^149^ Recent studies have revealed that GBM contacting the lateral ventricles disrupts SVZ ependymal cells via the Notch pathway, leading to reduced functional cilia, lipid droplets, and cerebrospinal fluid entry into tumor tissue.^40^^,^^150^ Moreover, GBM cells promote the selective migration of NSCs into primary GBMs and track invasive lesions throughout the brain by releasing soluble factors such as CXCL12, hypoxia-inducible factor-1 alpha (HIF-1α), platelet-derived growth factor (PDGF), and VEGF.^151^, ^152^, ^153^, ^154^

### Status quo of GBM therapies targeting the SVZ

As a highly malignant brain glioma, GBM is mainly treated by surgery, radiotherapy, chemotherapy, and targeted therapy. The SVZ has an extremely close relationship with GBM. Therefore, exploring GBM treatment regimens targeting the SVZ has been initiated. Here, we summarize the current GBM therapies designed to target the SVZ.

### Surgical treatment of GBM targeting SVZ

Currently, surgery remains the primary treatment for GBM, including total and subtotal resection. Maximal tumor resection is critical for treating and preventing GBM recurrence.^155^^,^^156^ Early studies show that the extent of resection of at least 78% or higher can improve overall survival, while for recurrent GBM, joint guidelines by the American and European Neuro-Oncology Societies indicate surgery may benefit symptomatic or larger lesions only if total resection is achieved. Preoperative MRI, ultrasound localization, *etc*. can expand the extent of resection, but their utility is often limited by brain anatomical displacement and compression.^156^ Since the ependyma is located on the inner side of the ventricular wall, preserving its integrity during SVZ-involved GBM resection surgery is challenging. Moreover, previous studies show that opening the ventricle intraoperatively increases risks of leptomeningeal dissemination, hydrocephalus, shortened survival, and other poor prognostic outcomes.^157^^,^^158^ Additionally, surgery can lead to permanent neurofunctional impairments. Given the infiltrative growth and ambiguous boundaries of SVZ-involved GBM, true complete pathological resection is difficult to accomplish surgically.

### Radiotherapy of GBM targeting SVZ

Currently, SVZ involvement is considered an independent prognostic factor for poor GBM outcomes. However, therapeutic effects of targeted SVZ radiotherapy vary, likely related to the dose, location, and volume of radiation.^159^^,^^160^ According to reports, SVZ radiation doses over 43Gy and 40Gy significantly prolong progression-free survival, indicating controlled radiation benefits GBM prognosis.^161^^,^^162^ The improvement of overall survival was more significant in patients receiving high-dose radiation to ipsilateral versus contralateral SVZ.^160^ In contrast, other reports indicate that there is no correlation between SVZ dose and progression-free survival or overall survival with either ipsilateral, contralateral, or bilateral radiation. More seriously, proposed high-dose SVZ radiation may provide radioresistant tumor stem cell survival space by eliminating NSCs, aggravating GBM progression.^163^^,^^164^

### Pharmacological treatment of GBM targeting SVZ

Since 2005, the oral alkylating agent temozolomide has been the first-line chemotherapy for GBM patients. As an adjuvant with radiotherapy, temozolomide can significantly prolong patient survival and improve quality of life,^165^ but most patients develop temozolomide resistance in later chemotherapy. This resistance is mainly associated with DNA repair systems like O^6^-methylguanine-DNA methyltransferase (MGMT)^166^ and mismatch repair in GBM cells, high GBM cell autophagy rates,^165^ and glioma stem cell phenotypic heterogeneity. However, temozolomide resistance related to SVZ is still unexplored. In recent developments, tumor-treating fields (TTFields) have been validated to enhance the prognosis of GBM patients when combined with temozolomide.^167^ Research indicates that TTFields not only suppress GBM cell proliferation but also result in a reduction of NSC markers and an increased expression of GFAP in normal brain organoids. This study suggests the potential for exploiting TTFields technology to target NSCs within the SVZ as an adjunct to chemotherapeutic strategies for GBM.^168^

### Targeted treatment of GBM via the SVZ

Current gene therapies are unable to infiltrate brain parenchyma and the core of GBM, while NSCs exhibit tumor tropism, laying the foundation for NSC-based targeted treatment of GBM. Induced NSCs (iNSCs), derived from skin fibroblasts, offer an autologous NSC treatment that can reduce immune rejection. Bagó et al first demonstrated that iNSCs possess tumor tropism similar to brain-derived NSCs and engineered mouse iNSCs to express a secreted variant of tumor necrosis factor-related apoptosis-inducing ligand (TRAIL; iNSC-sTR), finding that iNSC-sTR therapy has significant therapeutic effects on malignant GBM. In a subsequent phase I trial, they used early tumor-homing iNSCs (h-iNSCTE) loaded with the prodrug ganciclovir and TRAIL, showing that cytotoxic h-iNSCTE treatment can inhibit the progression and recurrence of GBM.^169^

Currently, the FDA-approved NSC line HB1.F3.CD, used for human clinical trials, can express cytosine deaminase (CD) and localize to GBM. HB1.F3.CD can convert the prodrug 5-fluorocytosine (5-FC) into the active chemotherapy drug 5-fluorouracil (5-FU), killing surrounding dividing tumor cells. Aboody et al showed that *in situ* GBM mice receiving HB1.F3.CD NSC (CD-NSC) and 5-FC combination treatment had significantly reduced tumor volumes compared with the control group.^170^ Data from the first human study by Portnow et al showed that CD-NSCs stopped dividing within 48 h after implantation and did not form secondary tumors, but continuous application was needed to maintain the anti-tumor effect.^171^ In a subsequent phase I clinical trial, Portnow et al determined the recommended phase II dose of oral 5-FC and calcium folinate for patients using CD-NSC therapy and demonstrated the feasibility of continuous intrathecal use of NSCs.^172^ Additionally, Ahmed et al and Fares et al used CD-NSCs loaded with engineered oncolytic adenovirus NSC-CRAd-S-pk7 for preclinical evaluation and the first phase I human trial, showing that NSCs loaded with oncolytic viruses could extend the survival of patients with various invasive GBM models and that continuous injection of NSC-CRAd-S-pk7 during surgery was relatively safe and tolerable for newly diagnosed GBM patients.^173^^,^^174^

Below are some other studies on targeted treatments for the SVZ. Matarredona et al used the CRISPR/Cas9 system to correct mutated tumor suppressor genes in SVZ NPCs, inhibiting the evolution of GBM.^112^ However, due to the multiple mutations in GBM, it is difficult to determine the driving mutations, and the feasibility of the CRISPR/Cas9 technique still needs to be verified. Research has shown that reducing the overall expression of GFAP in the SVZ is also a potential therapeutic target, as an increased GFAPδ/α ratio may activate genes involved in the interaction between GBM and the ECM, making GBM more invasive.^175^^,^^176^ Targeting telomerase in SVZ cells is also a promising targeted therapy. For example, adenine can down-regulate TERT to reduce telomerase activity and inhibit GBM proliferation.^177^ Also, some researchers used the specific C-X-C motif chemokine receptor 4 (CXCR4) antagonist AMD3100 (plerixafor) to treat U87MG mice, inhibiting SVZ-induced GBM invasion.^139^ Similarly, some researchers suggested adding fenofibrate as an adjuvant therapy to inhibit the migration of glioma stem cells to the SVZ and the proliferation of glioma stem cell precursor cells, NPCs, in the SVZ.^178^

GammaTile (γTile) is an FDA-approved surgical targeted radiotherapy (STaRT) for the treatment of recurrent GBM patients. It can be permanently implanted in the brain, providing patients with immediate, intense, and precise radiation after resection, reducing the recurrence rate at the treatment site and extending patient survival.^179^^,^^180^ However, current γTile therapy has limitations, such as difficulty controlling brain tumor cells more than 5–8 mm from the STaRT implantation cavity, and the safety of using STaRT in combination with temozolomide/external beam radiation therapy for newly diagnosed GBM has not been determined.^181^

In summary, regarding the treatment of GBM involving SVZ, although surgery, radiotherapy, and chemotherapy are currently the mainstream treatment methods, there are difficulties in achieving true pathological resection and resistance to radiotherapy and chemotherapy. Therefore, in the future, we believe that using NSC of SVZ as a carrier and utilizing its tumor propensity to load delivery enzymes/precursor drugs/viruses, such as tumor necrosis factor related apoptosis inducing ligands, precursor drugs such as ganciclovir, and oncolytic viruses, is a therapeutic approach that can address drug resistance and radiation therapy resistance. All in all, we believe that developing NSC as a delivery system to directly target the treatment of GBM affected by SVZ in the future is a key research focus.

## Conclusions and perspectives

In summary, the human SVZ holds significant implications in the repair of neural damage, the onset and progression of neurodegenerative diseases, and the resistance to glioma treatment. The SVZ can serve as a potential therapeutic target, offering novel insights and approaches for the treatment of brain injuries and degenerative diseases, and providing a scientific basis for neural stem cell therapy. SVZ cells have a broad application prospect, and current research still necessitates a deeper exploration of their biological characteristics and their application in the treatment of neurological diseases. However, we still need to address the issues of SVZ cell proliferation, differentiation, and migration mechanisms, explore their application in stem cell transplantation, gene therapy, and drug delivery, as well as overcome the problem of immune rejection, in hopes of providing new insights and methods for the treatment and prevention of neurological diseases. In summary, research on the SVZ is of great importance, promising to provide new insights and methods for the treatment and prevention of neurological diseases and to offer a scientific basis for neural stem cell therapy.

## Ethics declaration

This work was supported by the Ethics Committee of Nanfang Hospital, 10.13039/501100013853Southern Medical University (approval number: NFEC-2021-364). The rodent models utilized in Fig. 1 are derived from the project (A2022125) and undergo ethical scrutiny (approval number: IRB-2021-103).

## Author contributions

Ling Qi is the primary designer of this study. Kaishu Li, Yin Zheng, Shubing Cai, Zhiming Fan, and Junyi Yang contributed as the primary authors of the article. Siyuan Du, Yuanrun Liu, and Shengqi Liang performed the literature search. Meihui Song conducted a rigorous review of the content. All authors approved the final manuscript and submission.

## Funding

This work was supported by the 10.13039/501100003785Medical Scientific Research Foundation of Guangdong Province, China (No. A2022125, A2023486), the Medical Research Fund of the Qingyuan People's Hospital (No. 15001019002213), the National Natural Science Foundation of China (No. 82203351), and The Guangdong Basic and Applied Basic Research Foundation of China (No. 2021A1515111095).

## Conflict of interests

The authors declared no conflict of interests.
